# Identification of *Meloidogyne incognita* resistance QTLs using high density GBS linkage map in bittergourd

**DOI:** 10.3389/fpls.2026.1920925

**Published:** 2026-09-23

**Authors:** Shipra Thakur, Mamta Pathak, Navraj Kaur Sarao, Sukhjeet Kaur, Dharminder Pathak, Sukhpreet Kaur

**Affiliations:** 1 Department of Vegetable Science, Punjab Agricultural University, Ludhiana, Punjab, India; 2 School of Agricultural Biotechnology, Punjab Agricultural University, Ludhiana, Punjab, India; 3 Department of Plant breeding and genetics, Punjab Agricultural University, Ludhiana, Ludhiana, Punjab, India

**Keywords:** genotyping -by-sequencing, KASP markers, *Meloidogyne incognita* Momordica balsamina, Momordica charantia, QTL mapping

## Abstract

The root knot nematode (RKN; *Meloidogyne incognita*) is a very serious biotic constraint that limits the yield potential of bittergourd. The resistance within the cultivated germplasm of bittergourd is limited, therefore, introgressing resistance from related species of bittergourd offers a sustainable breeding solution. In this study, a RKN-susceptible cultivated parent *M*. *charantia* cv. Punjab-14 was crossed with resistant wild species of *M*. *balsamina* cv. Punjab Jhaar Karela-1 to generate F_2:3_ mapping population. A genetic linkage map was constructed via Genotyping-by Sequencing (GBS). Joint phenotyping and genotyping analysis identified a total of five quantitative trait loci (QTLs) governing RKN resistance. Under artificial screening, two QTLs namely QTLs *qRKN_pau6.1* (chromosome 6) and *qRKN_pau11.1* (chromosome 11) were detected, explaining 10.11% and 7.53% of phenotyping variance (PVE), respectively. Under field conditions, three QTLs namely, *qRKN_pau3.1*, *qRKN_pau6.2* and *qRKN_pau9.1* were mapped on chromosome 3, 6 and 9 accounting for 9.09%, 6.49% and 9.16% PVE respectively. The major artificial screening locus *qRKN_pau6.1* was successfully validated using kompetitive Allele-Specific PCR (KASP) assays. One diagnostic KASP marker, BGST_19012274 and one linked marker BGST_29234979 located within the resistance-associated genomic region, narrowed the genetic interval to 4.23 cM and 19.05 cM, respectively. To our knowledge, this is the first report on pathogen resistance introgression from *M*. *balsamina* into *M. charantia* and provides the first SNP-validated blueprint for marker-assisted selection against *M*. *incognita* in bittergourd.

## 1 Introduction

Bitter gourd (*Momordica charantia* L., 2x = 2n = 22), a member of the Cucurbitaceae family, is an economically vital vegetable crop cultivated extensively across tropical and subtropical regions (Basch et al., 2003; Asna et al., 2020). Renowned for its distinct bitterness, the species possesses high nutritional value and a well-documented history in traditional medicine (Gayathry and John, 2022). Molecular analyses indicate that eastern India serves as its primary center of origin, while China acts as a major secondary center of genetic diversity (Dey et al., 2006; Gaikwad et al., 2008; Kundu et al., 2012). Major cultivation and diversification of bittergourd exists in India, China, Malaysia, South America, and Africa (Miniraj et al., 1993). The crop thrives best in warm climate, making the summer season ideal for its cultivation and the immature fruits are harvested for optimal texture and palatability (Gayathry and John, 2022). Beyond its dietary role, *M. charantia* exhibits rich antioxidant, antihepatotoxic and anti-cancer properties driven by diverse bioactive phytochemicals, including triterpenoids, saponins, phenolics, and polypeptide-P (Semiz and Sen, 2007; Krishnaiah et al., 2011; Brennan et al., 2012; Han et al., 2025). Collectively, these characteristics underscore its significance not only as a horticultural crop but also as a functionally important medicinal plant with broad therapeutic potential (Hussain et al., 2024). Due to the medicinal properties, global consumer demand for bitter gourd has increased significantly (Warrier et al., 1997). However, its production is drastically threatened by bacterial, fungal, viral and nematode pathogens. Among these, the root-knot nematode (RKN; *Meloidogyne incognita* spp., Heteroderidae) stands out as one of the most destructive pests, capable of inflicting yield losses between 30% and 40% (Pandey and Nayak, 2018; Walia and Khan, 2023). Various efforts were made to study the transmission, causal pathogen, host range and etiological impact of the disease. Galling of roots is caused by nematodes belonging to genus *Meloidogyne* and family Heteroderidae (Walia and Khan, 2023). This group of nematodes causes severe characteristic galling of the roots which disrupt the water and nutrient uptake by the plants. Gall formation reduces the effective surface area for nutrient absorption and thus plants show wilting and chlorosis even when the soil moisture is adequate. Root-knot nematode cause losses in wide range of crops, including vegetables, fruits, ornamentals, pulses, cereals and oilseed crops (Caillaud et al., 2008). The disease symptoms include root galls, reduced and distorted root system, stunting, yellowing, reduced flowering, fruiting and overall yield decline. Secondary infections can also result from bacteria and fungi which often invade galled roots, causing root rot (López-Gómez and Verdejo-Lucas, 2014). The chemical methods used to control the RKN generally pollute the environment and cause toxicity to humans, animals and beneficial soil organisms and the control is short term (Khan et al., 2023). There limited sources of resistance available for root-knot nematode resistance in bitter gourd, however *Momordica balsamina*, (2n=2x=22) a related wild species of bittergourd also known as jhaar karela or vaarh karela, has been reported to carry complete resistance against root-knot nematodes and can be utilized for transferring the resistance to the cultivated bittergourd species (Sharma et al., 2019). One of the most sustainable ways of managing the RKN in bitter gourd is through host plant resistance. So, the development of improved disease-resistant cultivars should be the primary goal for the breeders. In 2017, PAU, Ludhiana released a stable, resistant Jhaar Karela cultivar namely “Punjab Jhaar Karela-1” for commercial cultivation (Pathak and Kaur, 2020).

But, introgressing this durable resistance into cultivated lines is restricted by severe pre- and post-zygotic reproductive barriers, including pollen-pistil incompatibility, embryo abortion, and genomic divergence. Despite these hurdles, a persistent 10-year wide-hybridization program at the Department of Vegetable Science, PAU Ludhiana, successfully generated interspecific hybrids, validated via SSR markers (Parameshwar, 2021). These interspecific hybrids displayed low seed set and a high frequency of non-viable hollow seeds resulting in only two surviving hybrid plants. Selfing of these individuals produced F_2_ segregating population, which was subsequently advanced to the F2:3 generation via single-seed descent to fix homozygosity.

Till date, no investigations have documented the identification of DNA markers or the construction of genetic maps associated with resistance to root-knot nematodes (RKN) in bitter gourd. Marker-assisted selection (MAS) is a powerful breeding strategy in which molecular markers closely linked to target traits enable breeders to minimize the need for extensive phenotypic screening, thereby saving time, labor and financial resources. Advances in next-generation sequencing (NGS) technologies have revolutionized marker discovery, enabling high-throughput genotyping approaches such as Restriction site-associated DNA sequencing (RAD-seq; Baird et al., 2008) and genotyping-by-sequencing (GBS; Elshire et al., 2011) to facilitate efficient single nucleotide polymorphism (SNP) detection, linkage map construction, and quantitative trait locus (QTL) identification in bitter gourd. Leveraging these tools, Urasaki et al. (2017) released the first scaffold-level genome assembly of the bitter gourd inbred line OHB3–1 and generated a linkage map comprising 11 linkage groups using RAD-seq derived markers. Further refinement was achieved by Cui et al, (2018), who also employed RAD-seq SNP markers to construct an improved linkage map, while Rao et al, (2018) utilized GBS in the absence of a reference genome to uncover SNPs for mapping applications. The most recent advancement in the genomic resources for bitter gourd is the publication of a chromosome-scale genome assembly, accompanied by an associated genetic linkage map, which represents one of the most comprehensive and well-resolved genomic assemblies currently available within the family Cucurbitaceae (Matsumura et al., 2014).

In this study, we used a reference-based GBS approach for SNP identification and genotyping of a mapping population segregating for root-knot nematode resistance. The identified SNPs were used in the construction of linkage map and detection of loci associated with RKN.

## 2 Material and methods

### 2.1 Plant materials

The *Momordica balsamina* cv. Punjab Jhaar Karela-1 (PJK-1), a root-knot nematode resistant parent and *Momordica charantia* L. cv. Punjab-14 (Pb-14), a susceptible parent, were used to generate the F_2:3_ mapping population. A systematic breeding program for Jhaar Karela was initiated in 2008, which resulted in the development of a stable and root-knot nematode resistant advance line (MB-08-03) by 2017. This advance line was released for commercial cultivation in Punjab and named as Punjab Jhaar karela-1 (Pathak and Kaur, 2020). In the present study, Punjab-14, an elite cultivar of bittergourd with green shiny fruits was found to be susceptible to root-knot nematodes was used as a recipient parent and Punjab Jhaar Karela-1 as the donor parent. Both parents showed diverse characteristics and were used to develop an F_2:3_ mapping population. The F_1_ plants were produced through hand pollination in the spring and rainy season of 2022. Those F_1_ plants were then subsequently self-pollinated to produce F_2_ generation during the spring season of 2023. Individual F_2_ plants were then sown and self-pollinated to generate F_2:3_ plant progenies during rainy season of 2023. Schematic representation of development of mapping population presented in **Figure 1**.

**Figure 1:**
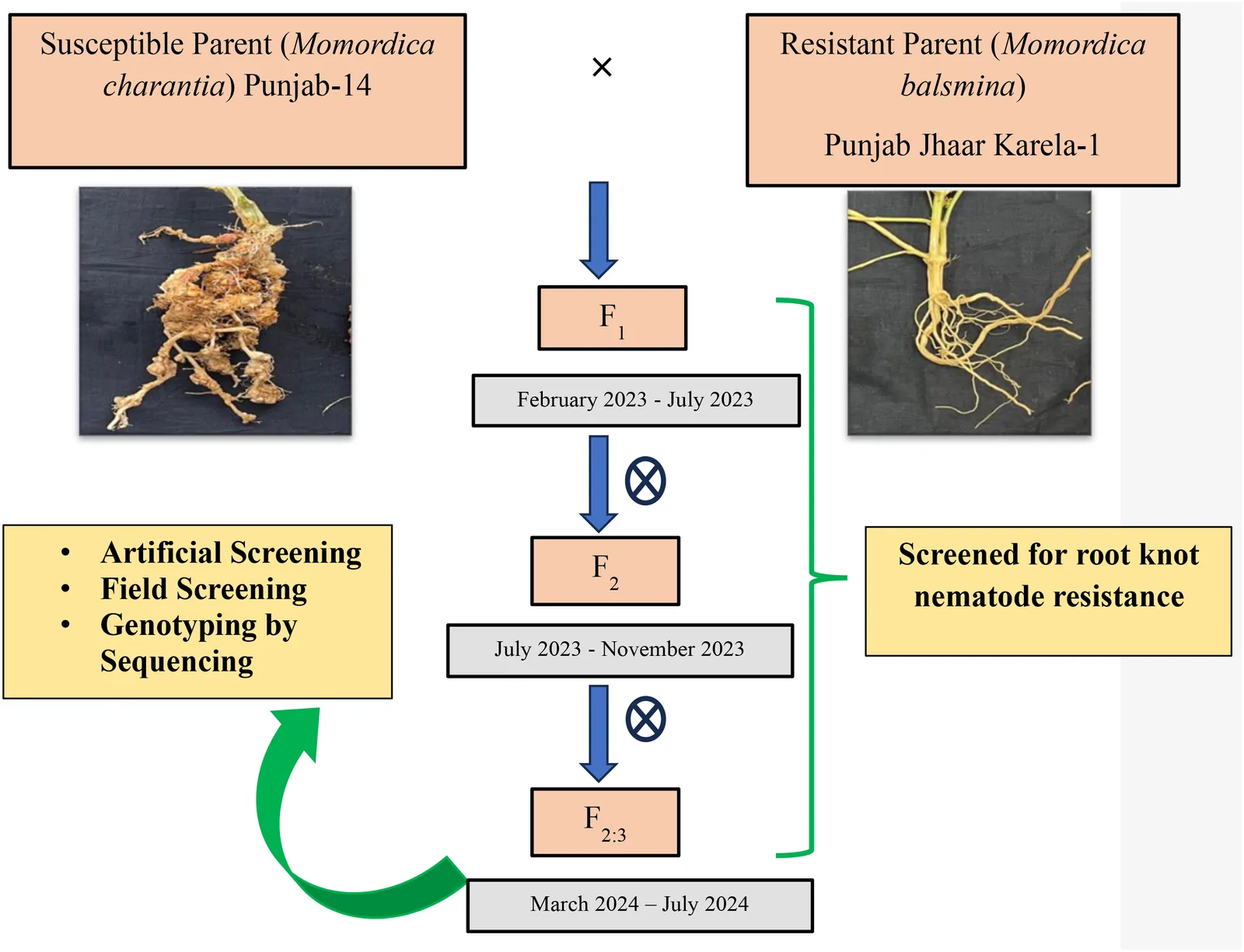
Schematic representation of the development and screening of the mapping population derived from the interspecific hybridization between *M. charantia* cv. Punjab-14 and *M. balsamina* cv. Punjab Jhhar Karela-1.

### 2.2 Pathogen isolation

The root-knot nematode culture used for screening was obtained from a pure culture of *Meloidogyne incognita* maintained on the RKN susceptible brinjal cultivar ‘Punjab Sadabahar’ grown in pots under greenhouse conditions. For inoculation, second-stage juveniles (J2) of *M. incognita* were extracted from infected roots as per Baermann funnel technique (Whitehead and Hemming, 1965). The infected brinjal plants were uprooted gently and infected roots were thoroughly washed, cut into small pieces (1–2 cm), and placed on double-layered tissue paper supported by a wire mesh in a Baermann funnel filled with distilled water, ensuring that the root material was just submerged. The setup was left undisturbed at room temperature (25 ± 2 °C) for 24–48 h to allow active juveniles to migrate from the root tissues into the surrounding water. The nematode suspension was then collected from the bottom of the funnel, concentrated, and the number of J2 was counted under a stereo zoom binocular microscope. A standard suspension containing 500 J2 per ml of water was prepared and used for further inoculations.

### 2.3 Phenotypic screening

The F_2_ population consisting of 151 plants was screened against root-knot nematode (*Meloidogyne incognita*) during the rainy season of 2023 under artificial conditions for the inheritance studies. For the development of mapping population F_2_ population was selfed to develop F_2:3_ mapping population. From the 151 F_2:3_ progenies, 118 individuals representing the F_2:3_ progenies were selected for the GBS genotyping. The seeds were sown in plastic plug trays containing a steam sterilized mixture of coco-peat, perlite and vermiculite in 3:1:1 ratio. Ten plants from each F_2:3_ progenies were sown in plug trays. At 3–4 leaf stage, seedlings were inoculated with 500 freshly hatched *M. incognita* juveniles per plant/plug. The suspension was poured into a small hole made near the plant base using a glass rod. Subsequently, roots were evaluated 28 days post-inoculation using a 0–5 scale given by Taylor and Sasser (1978), where score 0 (highly resistant): plant showed no galls; score 1 (resistant):1–2 galls; score 2 (moderately resistant): 3–10 galls; score 3 (moderately susceptible): 11–30 galls; score 4 (susceptible): 31–99 galls; score 5 (highly susceptible): >100 galls (**Table 1**). The screening procedure is shown in **Figure 2**. The plug trays were watered one day before taking observation. Plants were gently up-rooted and the roots gently tapped to clear the growing medium without causing any damage to the roots. Scores 0, 1 and 2 were pooled into resistant and scores 3, 4 and 5 into susceptible category for the inheritance study. After taking observation, seedlings were immediately transplanted into the grow bags filled with fresh potting mixture. The transplanted plants were then maintained properly for seed multiplication. Final nematode infestation scores were recorded at plant maturity. For field screening, a subset of 151 F_2:3_ families with 10 plants of each family were sown in separate trays without inoculation and were concurrently transplanted directly into the field during 2024 spring season to evaluate phenotypic response under natural conditions.

**Table 1:** Disease scoring scale for *Meloidogyne incognita* resistance given by Taylor and Sasser (1978).

Sr. no	Number of galls	Gall index (0-5)	Host susceptibility designation (Begum et al. (2014)
1	0	0	Highly Resistant
2	1-2	1	Resistant
3	3-10	2	Moderately Resistant
4	11-30	3	Moderately Susceptible
5	31-99	4	Susceptible
6	>100	5	Highly Susceptible

**Figure 2:**
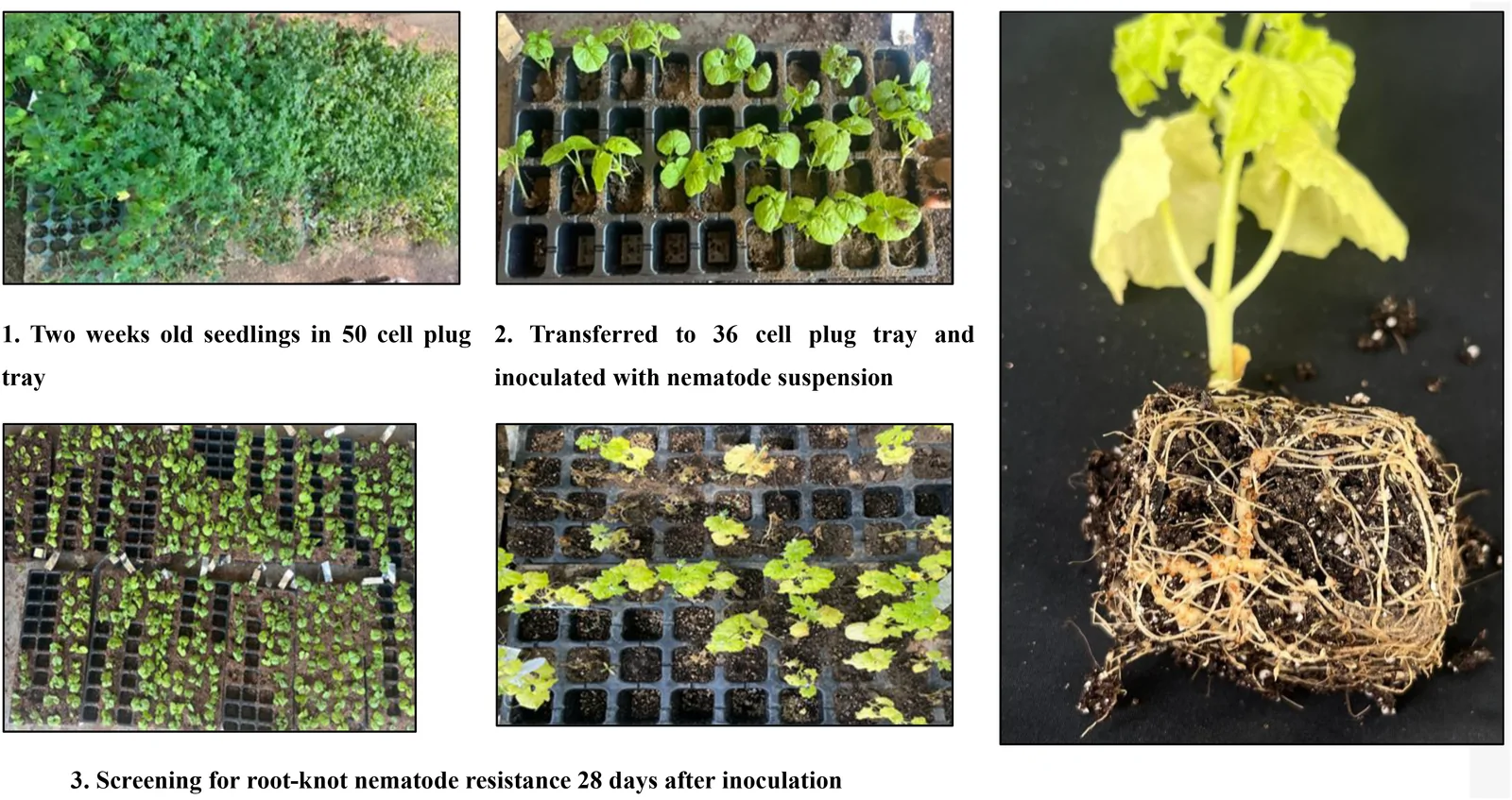
Schematic workflow of phenotypic screening steps for *Meloidogyne incognita* resistance in F_2:3_ mapping population in chronological order from sowing of the seeds in plug trays, artificial inoculation at 3–4 leaf stage, root scoring at 28 days post-inoculation and finally transplanting into grow bags for seed multiplication alongside field based screening.

### 2.4 DNA isolation, library construction and sequencing

Genomic DNA was extracted from young leaf tissues of both parental lines (Punjab-14 and Punjab Jhaar Karela-1) as well as 118 plants representing F_2:3_ progenies using a modified cetyltrimethylammonium bromide (CTAB) protocol (Doyle and Doyle, 1987) for genotyping purposes. The integrity and concentration of the isolated DNA were evaluated by electrophoresis on 0.8% agarose gels and quantified using a NanoDrop™1000 spectrophotometer (Thermo Scientific, Wilmington, USA), respectively. High-quality DNA samples were subsequently normalized to a concentration of approximately 100 ng µL^-1^ and selected for genotyping-by-sequencing (GBS). Library preparation and sequencing of the bitter gourd genome were performed by N2 Jenomics (India). The GBS library was constructed using the restriction enzyme Ape-KI and sequenced on the Illumina HiSeq™ X10 platform (Illumina^®^ Inc., San Diego, CA, USA) employing paired-end reads with a length of 160 bp.

### 2.5 Identification of single nucleotide polymorphism

The standard dDocent pipeline (Puritz et al., 2014) was employed for processing the paired-end (PE) sequencing reads generated from both parental lines and F_2:3_ individuals. Initial quality assessment of the raw reads was performed using FastQC (Andrews, 2010), followed by the removal of low-quality sequences and adapter contamination using Trimmomatic (version 0.39; Bolger et al., 2014). The resulting high-quality reads were aligned to the bitter gourd reference genome OHB3-1_genome_v2 (Urasaki et al., 2017), obtained from the Cucurbits database, using the Burrows-Wheeler Aligner (BWA v0.7.18) with the MEM algorithm (Li and Durbin, 2009). Sequence alignment map (SAM) files generated after alignment were subsequently converted to binary alignment map (BAM) format using SAMtools. Single nucleotide polymorphism (SNP) calling was carried out using the HaplotypeCaller module of the Genome Analysis Toolkit {GATK (version 4.5.0.0)}. Following variant detection, SNPs were subjected to stringent filtering using VCFtools (v0.1.16) to exclude insertion–deletion polymorphisms (InDels), variants with more than 25% missing data, read depth below 4, base quality scores less than 20, and minor allele frequency (MAF) ≤ 0.05, thereby retaining only high-confidence SNPs for downstream analyses. These SNPs were further refined by eliminating residual missing data, heterozygous loci, and monomorphic sites, resulting in 4,098 high-quality polymorphic SNPs. The final SNP dataset was converted into a parent-based ABH format and subsequently utilized for genetic linkage map construction.

### 2.6 Genetic linkage map construction

Genetic linkage mapping was prepared using the filtered SNP dataset within MapDisto software (version 1.7.7; Lorieux, 2012). To ensure high-quality map construction, individual SNP marker was first evaluated for segregation distortion against expected Mendelian ratios utilizing a chi-square (χ²) test. Markers demonstrating significant segregation distortion or an absence of linkage were excluded from further analysis. Following this quality control filtering, a subset of 4,098 highly reliable SNPs was retained for downstream map assembly. To establish chromosomal frameworks, the remaining markers were assigned to specific linkage groups (LGs) using a maximum recombination frequency threshold of 0.3 and a logarithm of odds (LOD) score of 3.0. Recombination frequencies were converted into genetic distances, expressed in centimorgans (cM), via the Kosambi mapping function to account for chromosomal interference. The final graphical visualization of the established linkage groups was generated using MapChart software (version 2.2; Voorrips, 2002).

### 2.7 Quantitative trait locus analysis

Quantitative trait loci (QTL) analysis for root knot nematode resistance was performed via composite interval mapping (CIM) using windows QTL Cartographer software version 2.5 (Silva et al., 2012). To ensure high statistical stringency, a QTL was considered significant only if it exhibited a logarithm of odds (LOD) score greater than 2.5. The QTLs explaining >10% of the phenotypic variance (PV) were considered the major effect QTL whereas as those having PVE <10% were considered minor effect QTLs associated with root-knot nematode resistance. QTL nomenclature followed a standardized convention: trait name_institution name_chromosome number and QTL order on the chromosome. For example, *qRKN_pau6.1* designates the first root-knot nematode resistance QTL identified on chromosome 6 by Punjab Agricultural University (pau).

### 2.8 KASP primer design and genotyping

Based on the linkage mapping results, sequences flanking the QTL *qRKN_pau_6.1* was utilized for the design of kompetitive allele-specific PCR (KASP) primers using Primer3+. KASP assays were performed in a total reaction volume of 4 µL, comprising 2 µL of diluted genomic DNA (30 ng mL^-^¹), 1.944 µL of KASP master mix, and 0.054 µL of KASP™ primer mix. Thermal cycling was carried out using a touchdown PCR protocol, consisting of an initial activation step at 95 °C for 12 min, followed by 10 touchdown cycles at 95 °C for 20 s, 64 °C for 1 min, and 72 °C for 15 s. This was followed by 35 amplification cycles of 95 °C for 10 s, 60 °C for 1 min, and 72 °C for 15 s, with a final extension at 72 °C for 5 min. Fluorescence signals were recorded using a TECAN Infinite F200 Pro microplate reader (Tecan Group Ltd.), and genotype calling was performed using KlusterCaller software (LGC Genomics LLC). Genotypic clusters were classified as homozygous (FAM or HEX) or heterozygous (FAM/HEX). For KASP validation, DNA from each parental genotype was included as a biological sample and each sample was analyzed in two technical replicates. 176 plants were randomly selected from the F_2:3_ progeny rows for KASP marker validation.

## 3 Results

### 3.1 Phenotypic evaluation of the mapping population

Symptoms such as characteristic galls and visible egg masses were observed on the roots. The parents of the mapping population showed contrasting phenotypic reactions for root-knot nematode (RKN) resistance: *Momordica charantia* (Punjab-14) exhibited complete susceptibility, whereas *Momordica balsamina* (Punjab Jhaar Karela-1) showed uniform resistance, confirming their suitability for inheritance analysis. The interspecific F_1_ hybrid plants displayed a susceptible reaction (**Figure 3**). In the F_2:3_ mapping population, the gall index ranged from 0 to 5 (**Figures 4a, b**).

**Figure 3:**
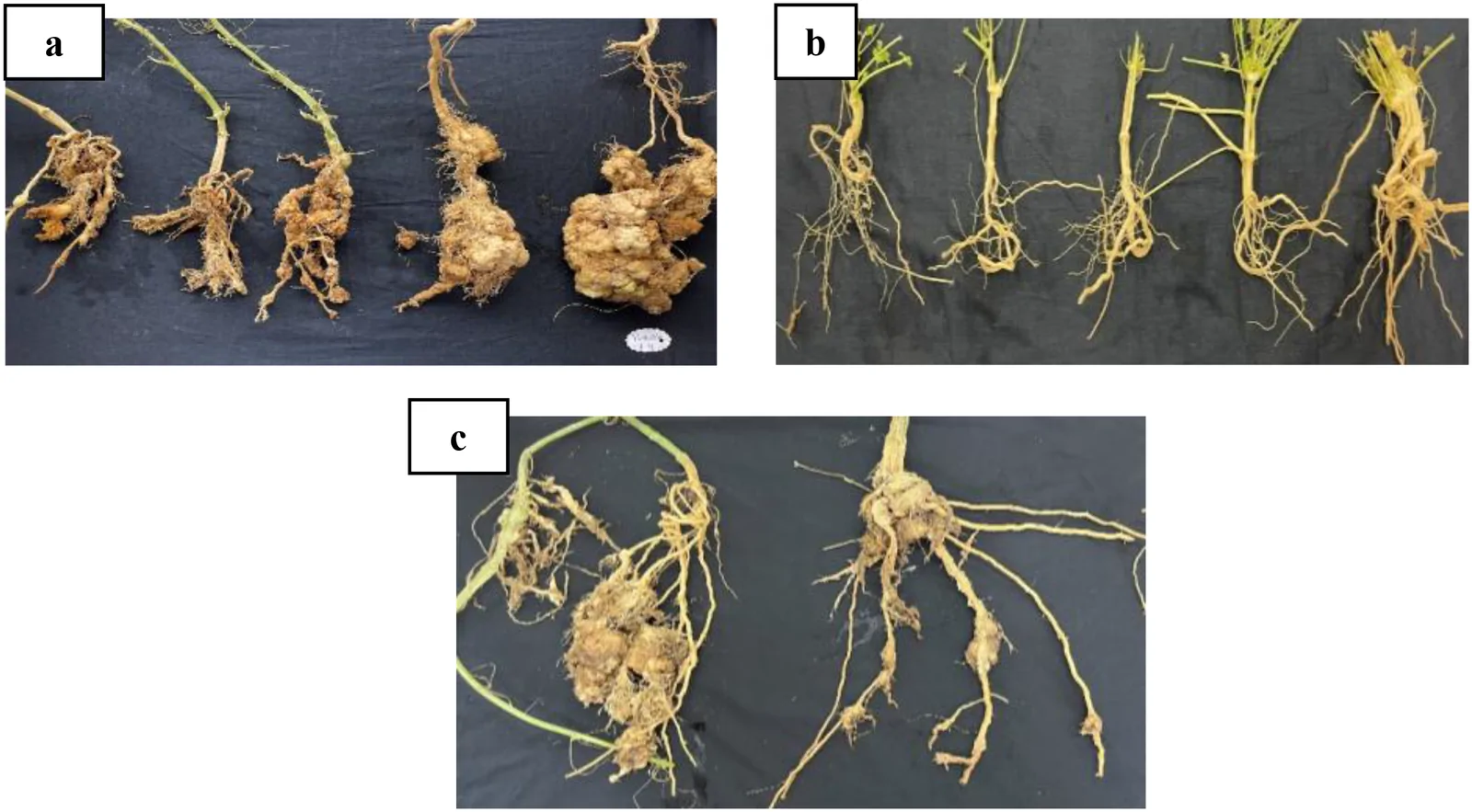
Pictorial representation of the contrasting phenotypes of the parental genotypes and F_1_ plants **(a)** Susceptible parent (*M.charantia* cv. Punjab-14) showing severe galling of the roots in response to the root-knot nematode infestation **(b)** Resistant parent (*M. balsamina* cv. Punjab Jhaar Karela) showing resistance against galling of the roots in response to the root-knot nematode infestation **(c)** Interspecific cross between *M.charantia × M.balsamina* (F_1_) showing severe galling of the roots in response to the root-knot nematode infestation.

**Figure 4:**
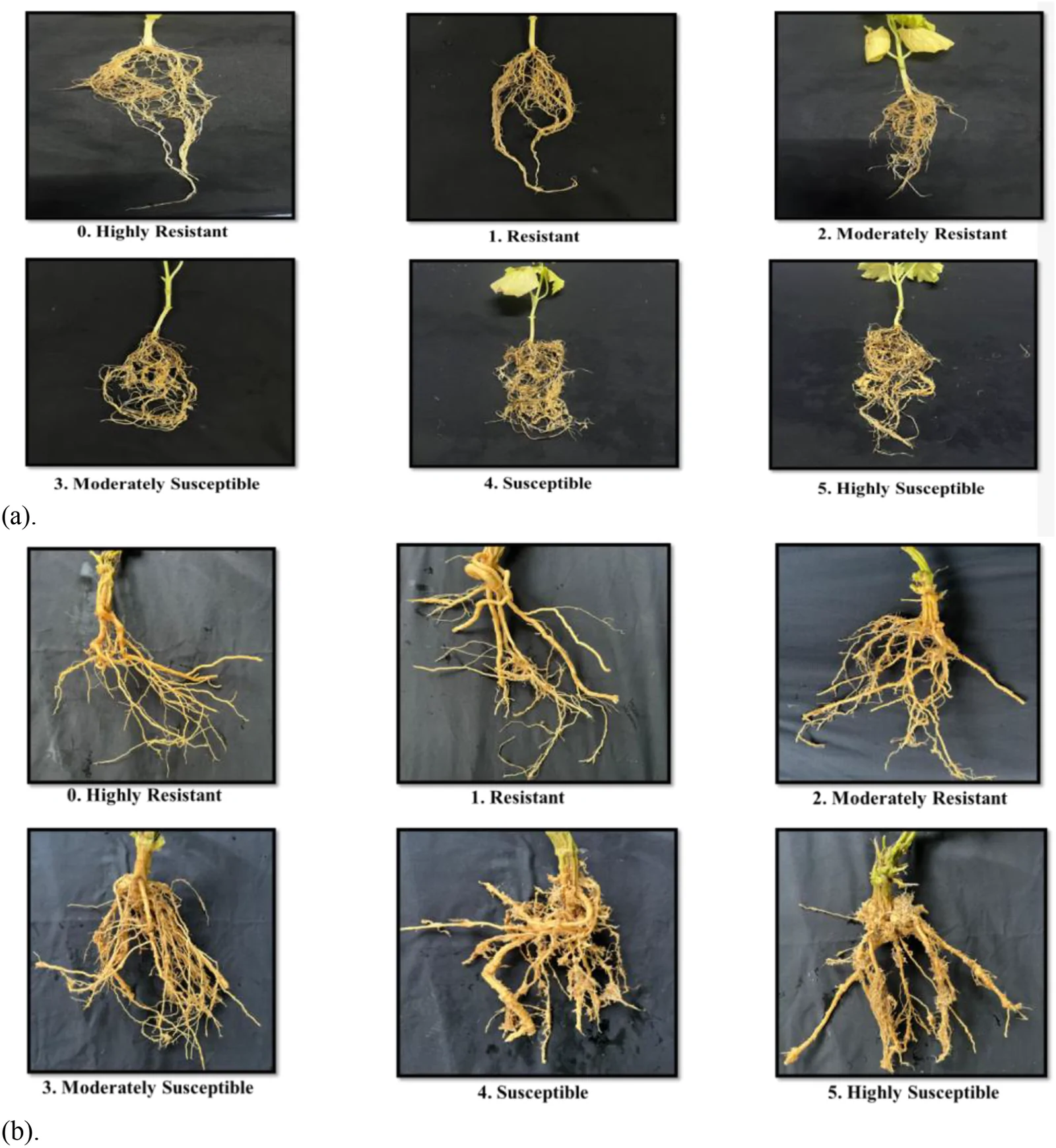
**(a)** Segregation pattern of the F_2:3_ population derived for interspecific cross between *M. charantia* cv. Punjab-14 × *M. balsamina* cv.Punjab Jhaar Karela-1 to the Meloidogyne incognita in the artificial screening method using 0–5 root gall index where (0) highly resistant (1) resistant (2) moderately resistant (3) moderately susceptible (4) susceptible (5) highly susceptible. **(b)** Segregation pattern of the F_2:3_ population derived for interspecific cross between *M. charantia* cv. Punjab-14 × *M. balsamina* cv.Punjab Jhaar Karela-1 to the Meloidogyne incognita in the field screening method using 0–5 root gall index where (0) highly resistant (1) resistant (2) moderately resistant (3) moderately susceptible (4) susceptible (5) highly susceptible.

Chi-square analysis elucidated the genetic control of RKN resistance in the Punjab-14 and Punjab Jhaar Karela-1 cross. In the F_2_ population (151 plants), segregation into resistant and susceptible classes fit a 3:1 (susceptible: resistant) ratio, as evidenced by a chi square value of 0.178, which was lower than the critical value at the 5% probability level (χ²^(0.05)^ = 3.84). This significant deviation strongly supports the hypothesis of monogenic inheritance for RKN resistance, suggesting that resistance to *Meloidogyne incognita* in bitter gourd is controlled by a single recessive gene (**Table 2**).

### 3.2 High-throughput sequencing and SNP identification

Sequencing of 118 F_2:3_ lines generated a total of 228.97 million paired-end (PE) reads, corresponding to 27.6 GB of data. The average sequencing depth at called genotype sites was 7.04× for Punjab-14 and 1.63× for Punjab Jhaar Karela-1, whereas the F_2:3_ progenies had a mean depth of 1.85 ± 0.15× (range: 1.41-2.25×). Thus, Punjab-14 showed substantially higher sequencing depth than the progenies, while PJK-1 had a depth comparable to that of the progeny population. Following quality trimming, the clean reads were aligned to the bitter gourd reference genome (OHB3-1_genome_v2). Alignment rates ranged from 95.58% to 98.94%, enabling reliable SNP discovery. A population size of 118 F_2:3_ individuals provided adequate power for the initial detection of major-effect QTLs, but the relatively small population may have limited the statistical power and mapping resolution for detecting and precisely estimating minor-effect QTLs, warranting validation in larger populations.

**Table 2:** Chi-square (χ2) analysis of F_2_ {*Momordica charantia* (Punjab-14) × *Momordica balsamina* (Punjab Jhaar karela-1)} showing inheritance pattern of RKN resistance.

Generation	Total number of plants	Disease reaction		Degree of freedom (df)	χ2 (cal)	χ2 _(0.05)_	P-value
		R	S				
P_1_ (Punjab-14)	20	–	10	–	–	–	–
P_2_ (PJK-1)	20	10	–	–	–	–	–
F_1_	2	–	2	–	–	–	–
F_2_	151 (Plants)	40	111	2	0.178 (3:1)	3.841	0.672

R, Resistant and S, Susceptible.

In total, 1,090,472 raw SNPs were identified across the 11 chromosomes, of which 467,205 were polymorphic. Subsequent filtering based on a minor allele frequency (MAF) threshold of 5% and a maximum of 25% missing data retained 72,299 SNPs. Further segregation distortion filtering using a chi-square test (p = 0.001) reduced the dataset to 4,098 high-quality SNPs (**Table 3**), which were subsequently employed for genetic linkage map construction. The chromosomal distribution of these SNPs across the 11 bitter gourd chromosomes is summarized in **Table 3**, and a Circos plot illustrating their genome-wide distribution is shown in **Figure 5**.

**Table 3:** Summary statistics of the bittergourd genetic linkage map constructed using F_2:3_ individuals derived from interspecific cross between *M. charantia* cv. Punjab-14 and *M. balsamina* cv. PJK-1.

Chr	Linkage group	No. of markers	Total genetic distance(cM)	Av. spacing (cM)	Max. spacing (cM)
**1**	1	330	796.91	2.42	39.47
**2**	2	253	519.66	2.06	43.85
**3**	3	297	828.57	2.71	47.81
**4**	4	487	1190.89	2.45	29.30
**5**	5	356	949.85	2.68	37.92
**6**	6	478	2294.75	4.81	33.48
**7**	7	263	807.23	3.08	34.81
**8**	8	479	1113.05	2.33	40.02
**9**	9	469	1066.52	2.28	33.77
**10**	10	345	988.64	2.87	42.34
**11**	11	341	944.00	2.78	38.62
Overall		**4098**	11500.07	2.77	47.81

**Figure 5:**
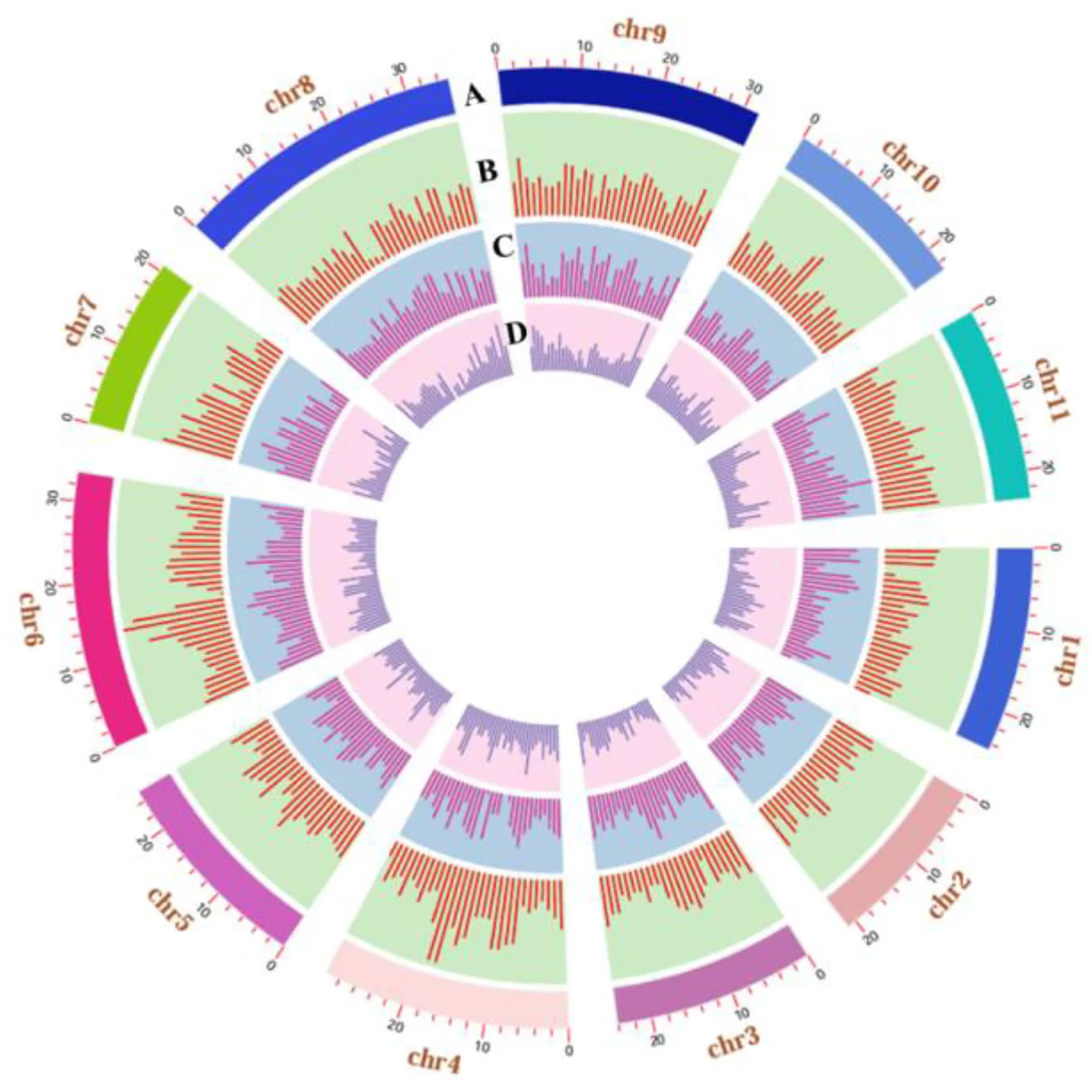
Distribution of SNPs identified in the F_2:3_ population from cross between *M. charantia* cv. Punjab-14 × *M. blsamina* cv. Punjab Jhaar Karela-1 in bittergourd. Track A represents 11 chromosomes in different colors. Track B, C and D represents raw SNPs, filtered SNPs and mapped SNPs within 100 kb window on 11 chromosomes.

### 3.3 SNP-based linkage map construction

A total of 4,098 SNP markers were successfully mapped, resulting in the formation of 11 linkage groups (LGs) corresponding to the 11 bitter gourd chromosomes. Collectively, the linkage groups covered a cumulative genetic distance of 11,500.07 centimorgans (cM). The genetic length of individual linkage groups ranged from 519.66 cM for LG2 to 2,294.75 cM for LG6. SNP markers were uniformly distributed across the linkage map, with each linkage group comprising between 253 and 487 SNPs (**Table 3**). The maximum observed inter-marker distance was 47.81 cM. The constructed linkage map, depicted in **Figures 6a and b**, illustrates the genomic organization of high-confidence polymorphic SNP markers across the 11 bitter gourd chromosomes, with marker positions represented in cM. The QC analysis was performed to check the quality of selected markers to evaluate the reliability of the final map.

**Figure 6:**
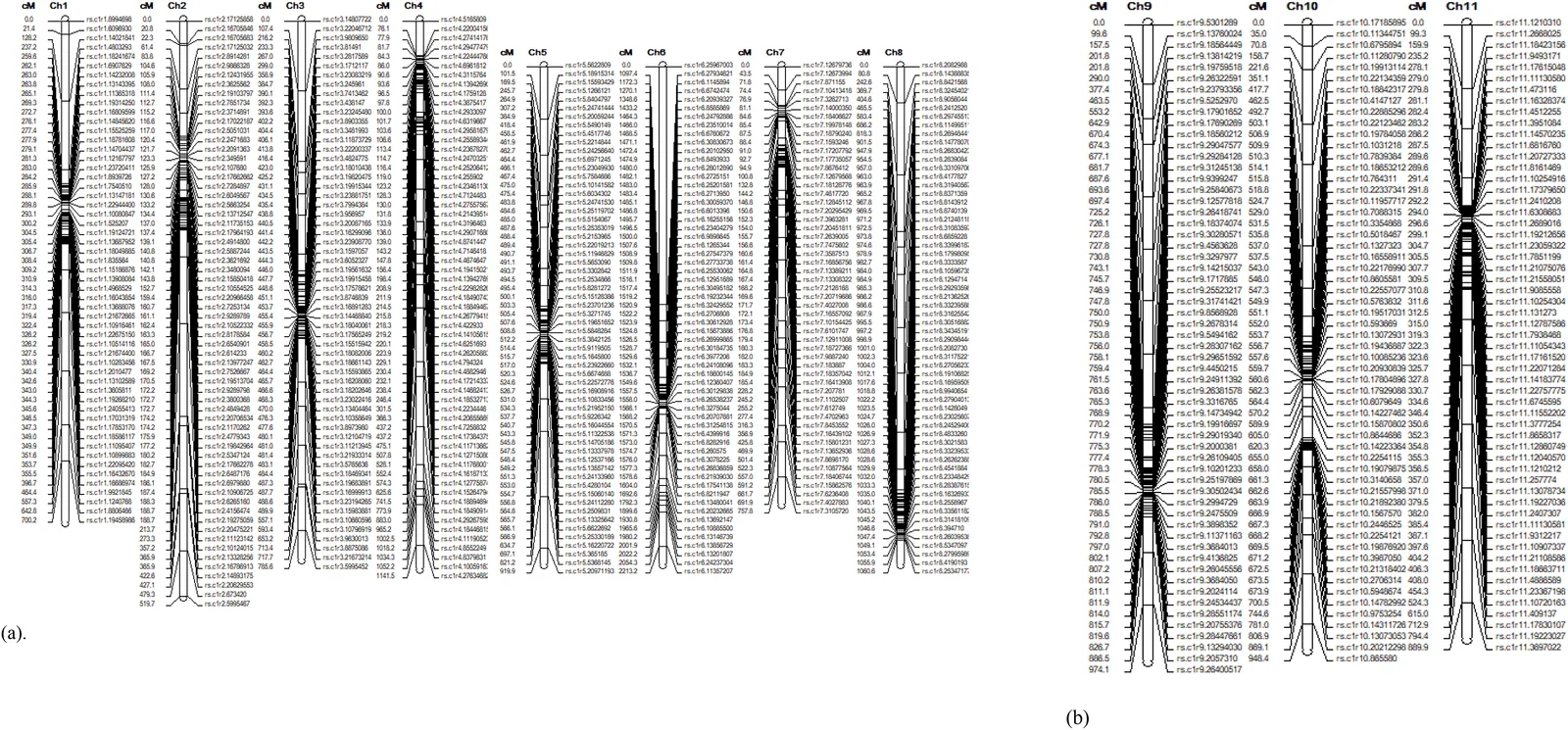
**(a)** Genetic linkage map developed from the F_2:3_ population derived from cross between *M. charantia* cv. Punjab-14 and *M. balsamina* cv. Punjab Jhaar Karela-1 (Chromosome 1 to 8). **(b)** Genetic linkage map developed from the F_2:3_ population derived from cross between *M. charantia* cv. Punjab-14 and *M. balsamina* cv. Punjab Jhaar Karela-1 (Chromosome 9 to 11).

### 3.4 Detection of QTLs conferring root-knot nematode resistance

Quantitative Trait Loci (QTL) analysis was performed using the generated linkage maps integrated with the corresponding root-knot nematode gall index phenotypic data from the F_2:3_ populations. Disease data from two separate trials i.e. artificial inoculation and field screening were analyzed using Windows QTL Cartographer software. A total of five QTLs for root-knot nematode resistance were identified on chromosomes 3, 6, 9, and 11 (**Table 4**; **Figure 7**).

**Table 4:** Quantitative trait locus identified for root-knot nematode resistance in bittergourd.

Environment	QTL name	Chromosome	Position (cM)	LOD score	PVE (%)	Flanking markers	CI (cM)
Artificial screening, 2024	*qrkn_pau_6.1*	6	2095.7	5.1	10.11	rs.chr6. 31007121 - rs.chr6. 31856674	17.8
	*qrkn_pau_11.1*	11	15.01	3.8	7.53	rs.chr11. 473048 - rs.chr11. 1210310	14
Field screening, 2024	*qrkn_pau_3.1*	3	483.11	4.5	9.09	rs.chr3. 5765636 – rs.chr3. 23279450	0.4
	*qrkn_pau_6.2*	6	1172.11	3.3	6.49	rs.chr6. 1145894 - rs.chr6. 6760768	3.3
	*qrkn_pau_9.1*	9	294.21	4.6	9.16	rs.chr9. 26322477 - rs.chr9. 26336372	4.6

**Figure 7:**
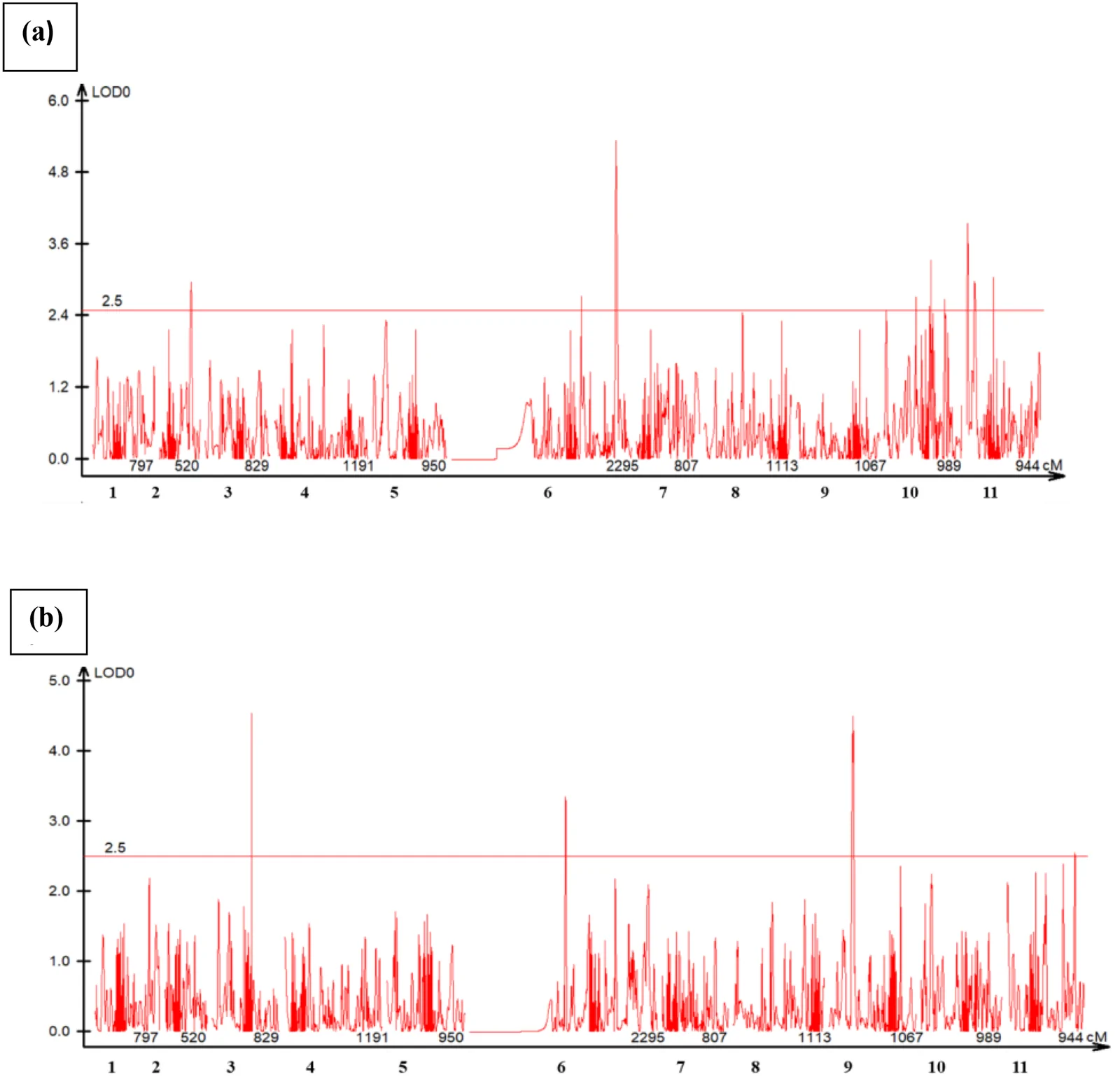
Quantitative trait locus detected for root-knot nematode resistance in **(a)** artificial screening and **(b)** field screening during the year 2024. Plots indicate genetic coordinate on the x-axis and LOD score on the y-axis of the identified QTLs.

The QTLs designated as *qRKN_pau6.1* and *qRKN_pau11.1* were identified via artificial screening. Locus *qRKN_pau6.1* explained 10.11% of the phenotypic variance (PVE) at a LOD score of 5.1 and exhibited an additive effect of -0.52. This QTL was located at 2095.7 cM within a genetic interval of 17.8 cM, flanked by markers rs.chr6.31007121 and rs.chr6.31856674. Another QTL *qRKN_pau11.1* was located on chromosome 11 at 15.01 cM and was flanked by markers rs.chr11.473048 and rs.chr11.1210310. This QTL showed a LOD score of 3.8, explaining 7.53% of the PVE.

Field screening revealed three additional QTLs on chromosomes 3, 6, and 9. Locus *qRKN_pau3.1* was situated at 483.11 cM between the flanking markers rs.chr3.5765636 and rs.chr3.23279450. Locus *qRKN_pau6.2* flanked by markers rs.chr6.1145894 and rs.chr6.6760768 on chromosome 6 at 1172.1 cM, explained 6.49% of the PVE at a LOD value of 3.3. Notably, chromosome 6 had QTLs common to both artificial and field screenings, though they occupied distinct genomic positions with different flanking markers. Lastly, *qRKN_pau9.1* on chromosome 9 was identified between flanking markers rs.chr9.26322477 and rs.chr9.26336372 at 294.21 cM, with a LOD score of 4.6 explaining 9.16% of the phenotypic variance. Physical (bp) positions of the reported QTL intervals relative to the reference genome assembly (OHB3-1_genome_v2) has been mentioned in **Supplementary Table 1**. The identified QTLs and important loci on the genetic map are shown in **Figure 8**, color-coded to reflect their associations with the two screening conditions.

**Figure 8:**
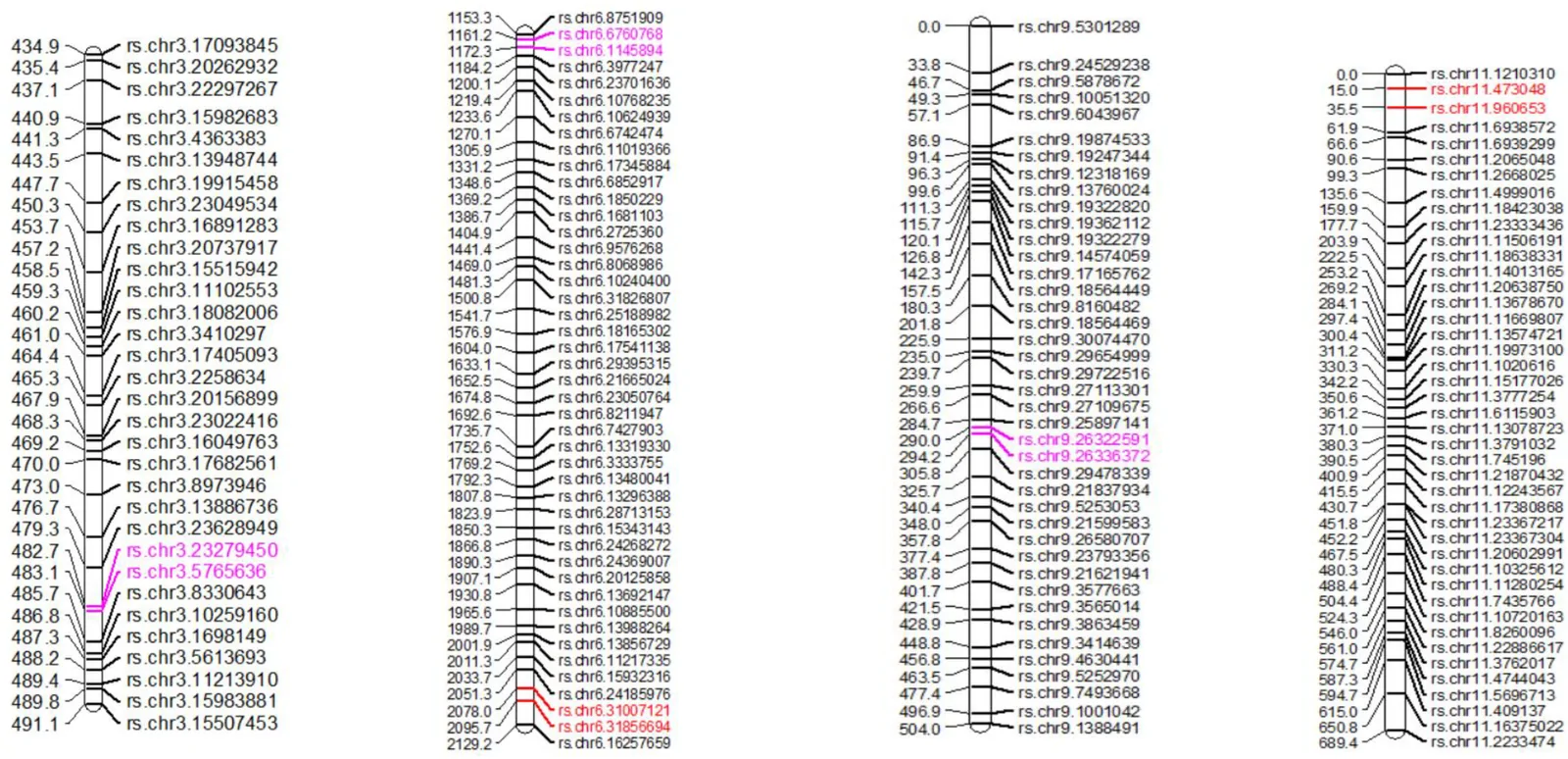
QTLs identified in artificial screening are indicated in red color and QTLs identified during filed screening are shown in pink color associated with the *Meloidogyne incognita* resistance in bittergourd.

### 3.5 KASP marker development and linkage mapping of the candidate region

Single nucleotide polymorphisms (SNPs) located within the *qRKN_pau6.1* interval were exploited to develop Kompetitive Allele-Specific PCR (KASP) markers to further validate the association of this QTL region with root-knot nematode resistance. KASP assays were designed using contrasting SNPs within the flanking marker interval that exhibited polymorphism between the resistant and susceptible parental genotypes. Primer sequences were carefully optimized to ensure chromosome 6 specificity and to avoid off-target amplification. KASP markers derived from both intronic and exonic regions of putative candidate genes were subsequently employed for classical QTL validation. A complete list of KASP markers developed for the *qRKN_pau6.1* region is provided in **Supplementary Table 2**.

Among the eleven KASP markers designed, five markers i.e. BGST_2659558, BGST_29234979, BGST_27733738, BGST_19012724, and BGST_30088637 displayed clear polymorphism between the parental lines (**Figure 9**). Of these, two KASP markers were selected to genotype the F_2:3_ population to validate their linkage with root-knot nematode resistance in bitter gourd (**Figure 10**). The resulting linkage map derived from F_2:3_ genotyping is presented in **Figure 11**. A genetic linkage map spanning 23.28 cM was constructed using these two KASP markers. The markers BGST_19012724 and BGST_29234979 were positioned at genetic distances of 4.23 cM and 19.05 cM, respectively, from the qRKN_pau6.1 locus. Statistical association between marker genotypes and resistance phenotypes (ANOVA) has been demonstrated for both markers in **Supplementary Table 3**. The phenotype-genotype association analysis of the two KASP markers revealed that BGST_19012724 showed a highly significant association with RKN resistance (F = 127.49, P = 3.08 × 10^-^²², η² = 0.437), indicating a strong relationship between marker genotype and resistance phenotype. In contrast, BGST_29234979 did not show a significant association (F = 0.61, P = 0.435, η² = 0.0045).

**Figure 9:**
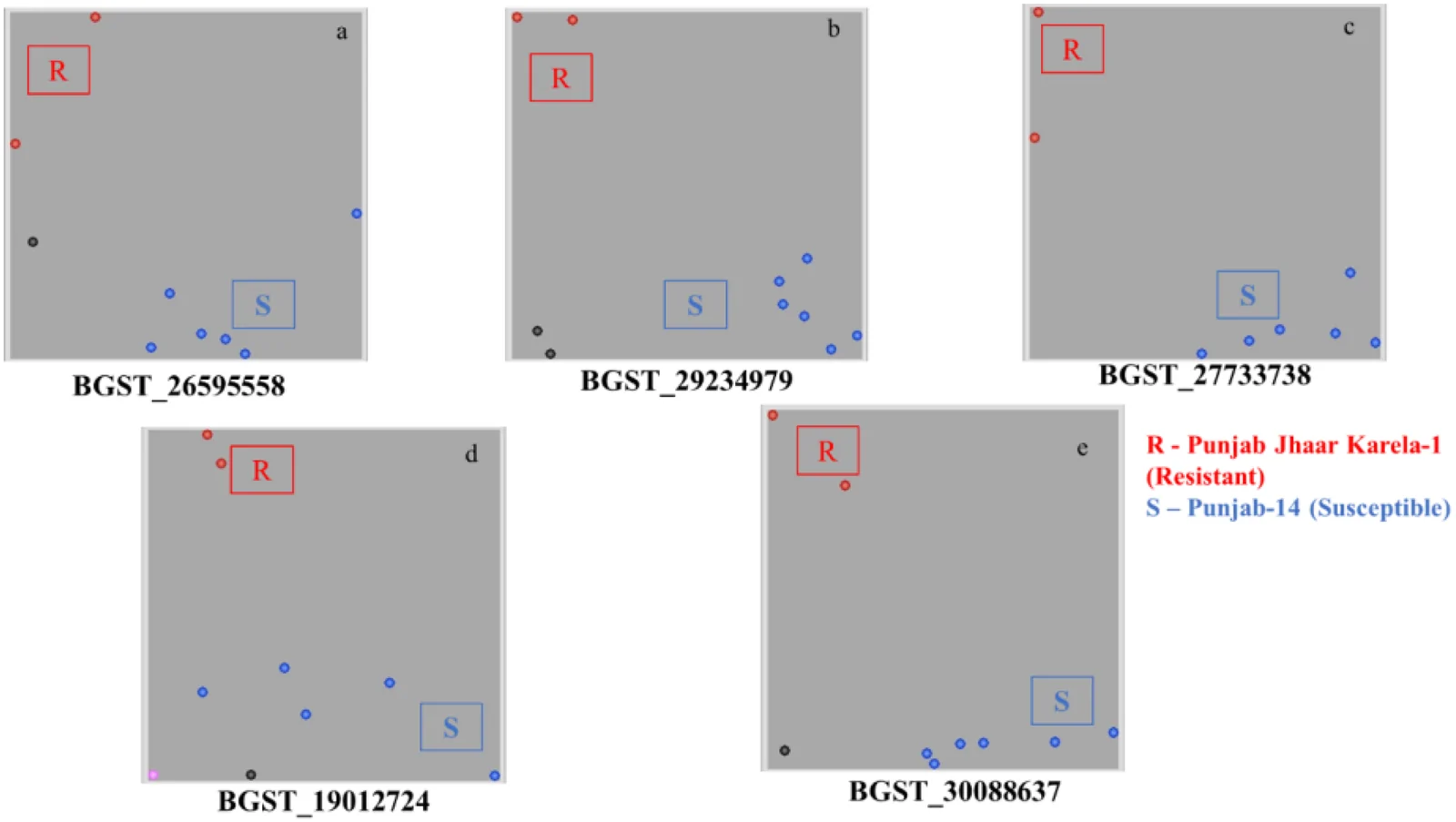
KASP assay cartesian cluster plot for parental polymorphism for root-knot nematode resistance in bittergourd. Panels a-e represent the clustering patterns for five polymorphic KASP markers developed from genomic region on chromosome 6. Five KASP markers are **(a)** BGST_26595558, **(b)** BGST_29234979, **(c)** BGST_27733738, **(d)** BGST_19012724 and **(e)** BGST_30088637. Red and blue dots indicate the two parental lines (PJK-1 and Pb-14) and black dots represents to the non-template control (NTC).

**Figure 10:**
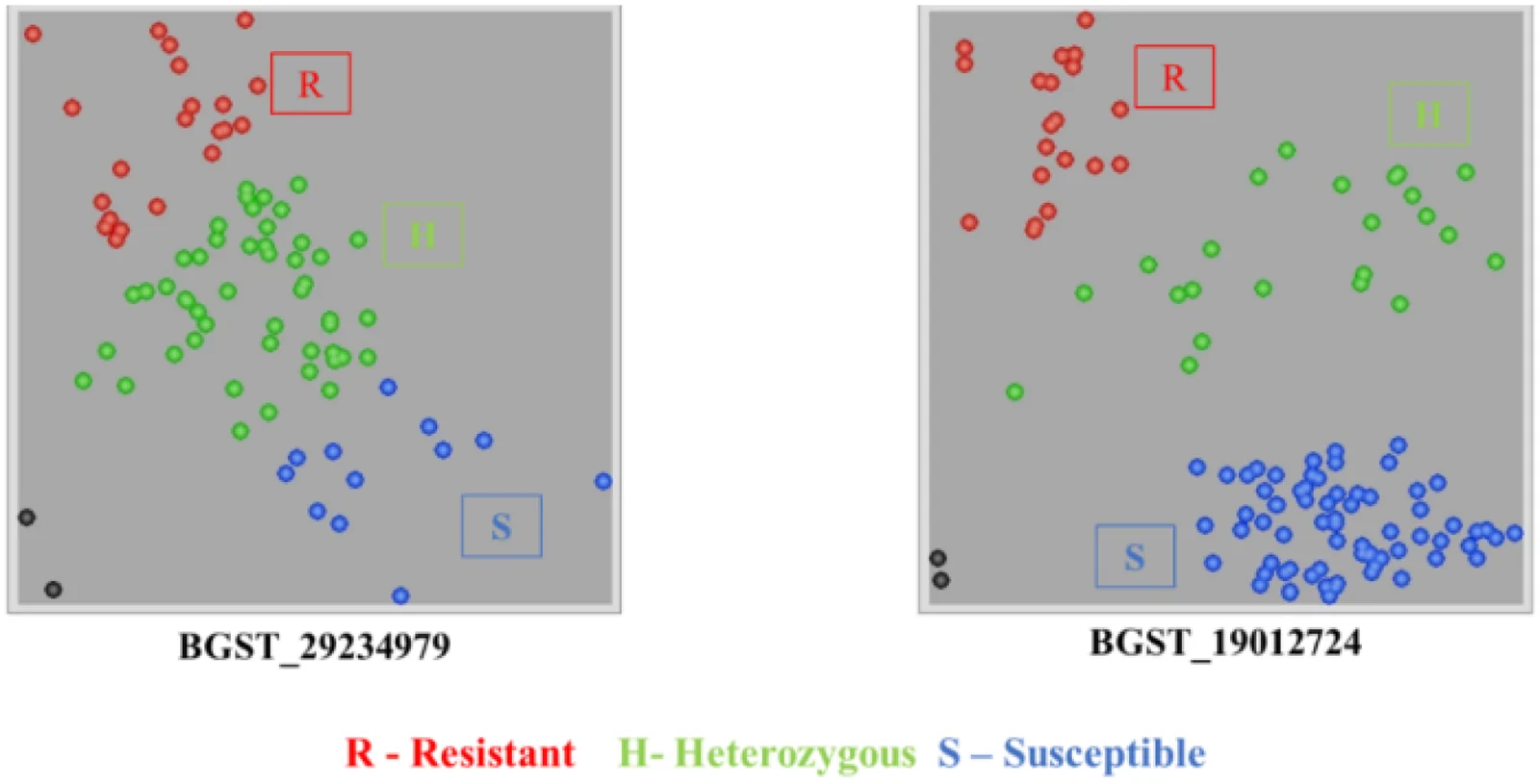
KASP assay cartesian cluster plot of 176 biparental F_2:3_ population for two KASP markers (i) BGST_29234979 and (ii) BGST_19012724. Red and blue dots represents homozygous F_2:3_ individuals and parents and green dots corresponds to heterozygous F2:3 individuals and black dots correspond to non-template control (NTC). .

**Figure 11:**
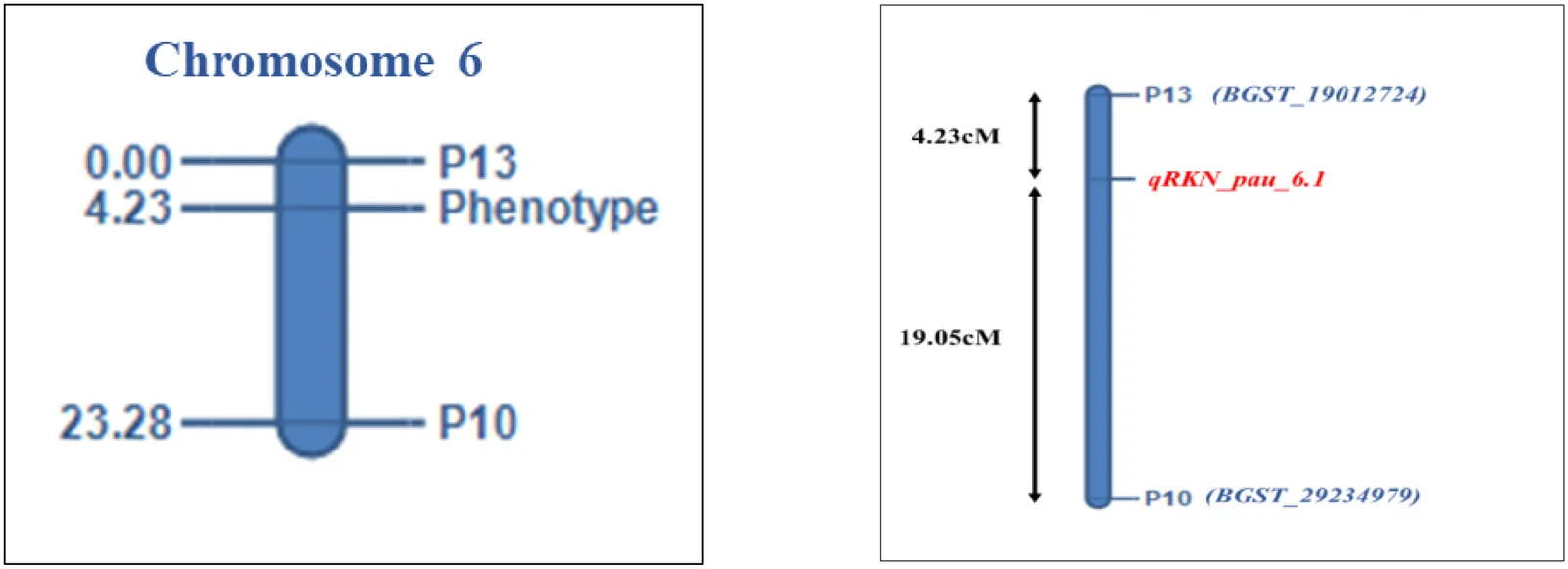
Genetic linkage map developed using the polymorphic KASP marker in F_2:3_ population (i) location and (ii) interval of the QTL region and the KASP markers associated with the root-knot nematode resistance trait in bittergourd.

In the present study, classical mendelian analysis of the F_2_ population (Section 3.1) based on categorial ratings of resistant or susceptible indicated that root-knot nematode (RKN) resistance is governed by a single recessive gene. However, QTL analysis (Section 3.4) using a quantitative rating scale revealed a multigenic genetic architecture. Specifically, *qRKN_pau6.1* was identified as a major effect QTL (explaining 10.11% PVE with a high LOD score of 5.1) associated with the recessive resistance phenotype. The remaining four minor QTLs *qRKN_pau3.1*, *qRKN_pau6.2*, *qRKN_pau9.1*, and *qRKN_pau11.1* likely act as genetic modifiers or minor-effect loci that modulate the expressivity of the gall index across varying environmental conditions.

To further investigate the genomic basis of resistance to *Meloidogyne incognita*, the genes annotated within the *qRKN_pau6.1* candidate region were examined for putative functions associated with plant defense and stress responses. A total of 20 annotated genes were identified within the investigated interval, of which several encoded proteins with potential roles in pathogen perception, defense signaling, transcriptional regulation, reactive oxygen species (ROS) homeostasis, secondary metabolism and cell-wall reinforcement (**Supplementary Table 4**). Among these, *Moc06g40190*, encoding an MDIS1-interacting receptor-like kinase 2-like protein, was identified as a potential pathogen-recognition/signaling component. Additional protein kinase genes, including *Moc06g40100, Moc06g40510* and *Moc06g40620*, were also present in the interval and may participate in phosphorylation-dependent defense signaling.

Several transcription factors with potential roles in defense regulation were identified, including *Moc06g39650*, encoding a bHLH126-like transcription factor, *Moc06g40420*, encoding a MYB domain-containing protein-like, and *Moc06g40450–Moc06g40470*, encoding AP2/ERF- and ethylene-responsive transcription factors. These genes are of particular interest because transcription factor families such as MYB, bHLH and ERF are extensively involved in transcriptional reprogramming during root-knot nematode interactions.

The candidate region also contained genes associated with phenylpropanoid metabolism and ROS-mediated defense. *Moc06g40350* encodes a peroxidase, while *Moc06g39710*, *Moc06g40040* and *Moc06g40050* encode cytochrome P450 proteins. In addition, *Moc06g40020* and *Moc06g40030* encode amine oxidases that may contribute to hydrogen peroxide production, and *Moc06g39760* and *Moc06g40440* encode phytocyanin-domain-containing proteins potentially associated with redox processes.

Notably, Moc06g40420, encoding a MYB domain-containing protein-like, represents one of the most interesting candidates because of its putative role in phenylpropanoid, flavonoid and lignin biosynthesis.

## 4 Discussion

Root-knot nematodes (RKN; *Meloidogyne* spp.) represent the most economically damaging pest limiting bitter gourd (*Momordica charantia* L.) production globally. Invasion by *M. incognita*, *M. javanica*, and *M. arenaria* induces root galls that disrupt vascular architecture, and disturbing water and nutrient translocation. The resulting nematode-induced giant cells act as metabolic sinks that reduces photosynthetic efficiency, overall plant vigor, flowering, and yield. Locally, *M. incognita* is highly prevalent, severely reducing both crop yield and quality. In India alone, this pathogen accounts for an estimated 30% to 40% reduction in bitter gourd yields (Pandey and Nayak, 2018). Consequently, a genetic study to determine the inheritance of root-knot nematode resistance in bitter gourd, followed by genetic mapping of these defense genes, is a critical prerequisite for targeted marker-assisted selection and durable cultivar breeding.

In this study, we evaluated an interspecific mapping population derived from a cross between the highly susceptible *M. charantia* line (Punjab-14) and a uniformly resistant *M. balsamina* wild relative accession (Punjab Jhaar Karela-1). Our phenotypic screening demonstrated a clear binary demarcation in parental reactions, confirming the genetic divergence of the chosen parents and establishing a robust framework for inheritance analysis.

Classical Mendelian analysis of the F_2_ population revealed a 3:1 susceptible-to-resistant segregation ratio (χ² = 0.178). This pattern demonstrates that a single recessive gene controls the primary restriction of nematode galling and reproduction in Punjab Jhaar Karela-1. This is in consistent with the study showing monogenic recessive control by mj gene in the cucumber line LJ 90430, providing strong resistance against *M. javanica* (Walters et al., 1997). However, complete reliance on Mendelian ratios can mask the minor-effect loci that fine-tune crop defense system. As the exact molecular mechanisms underlying RKN resistance in bitter gourd remain largely uncharacterized, integrating advanced genomic tools is essential for identifying evolving RKN strains and establishing durable management solutions.

While GBS is well-established in agronomic crops like cereals and cotton, its application in vegetable breeding is relatively recent. In cucurbits, GBS has successfully facilitated linkage mapping and QTL discovery for traits such as gynoecy, earliness, fruit quality, and yellow mosaic disease resistance across watermelon, bottle gourd, squash, muskmelon, and pumpkin (Meru and McGregor, 2016; Wu et al., 2017; Rao et al., 2021; Kaur et al., 2021, 2022). However, our study represents the first definitive report of mapping RKN resistance in bitter gourd.

By mapping an F_2:3_ population derived from the interspecific cross *M. charantia* cv. Punjab-14 (susceptible) × *M. balsamina* cv. Punjab Jhaar Karela-1 (resistant), we generated 228.97 million high-quality reads. A high alignment rate (95-99%) against the reference genome (Urasaki et al., 2017) confirmed data reliability. Stringent filtering of 1,090,472 raw SNPs for missing genotypes, minor allele frequency (MAF), and segregation distortion yielded 4,098 high-quality SNPs. Such a reduction in marker number is typical for GBS workflows (Zhang et al., 2015; Kaur et al., 2021). The resulting map covers a total genetic distance of 11,500.07 cM across 11 linkage groups, with a mean marker interval of 2.77 cM with a marker density of roughly 0.36 markers/cM. The previous bitter gourd and cucurbit frameworks, range from 0.30 to 1.30 markers per cM (Matsumura et al., 2014; Rao et al., 2018; Kaur et al., 2021). The exceptionally extended genetic map observed in the present study may reflect the combined effects of interspecific genomic divergence between *M. charantia* and *M. balsamina* and residual GBS-associated genotyping errors. Sequence divergence may increase allele dropout and genotype uncertainty, potentially generating spurious recombination events and apparent double crossovers. Such genotyping errors are known to inflate genetic distances and distort marker ordering (Hackett and Broadfoot, 2003; Cartwright et al., 2007), and are particularly relevant to GBS-based linkage mapping (Taniguti et al., 2023). Extended genetic maps have also been reported in interspecific mapping populations, supporting the possibility that divergent parental genomes can contribute to unusually large genetic distances (Wei et al., 2020; Kaur et al., 2025).

To counteract the challenges of monoecious flowering and high cross-pollination in bitter gourd, phenotyping was rigorously executed on F_2:3_ lines under both controlled artificial inoculation and natural field conditions. Phenotypic variation across trials underscored the impact of seasonal environmental fluctuations and pathogenic variability on QTL detection. QTL mapping revealed five genomic regions (*qRKN_pau3.1*, *qRKN_pau6.1*, *qRKN_pau6.2*, *qRKN_pau9.1*, and *qRKN_pau11.1*) on chromosomes 3, 6, 9, and 11, explaining 6.49% to 10.11% of the phenotypic variance. Notably, *qRKN_pau6.1* identified during artificial screening method emerged as the major determinant of resistance. It exhibited the highest LOD score of 5.1 and explained 10.11% of the phenotypic variance (PVE), with a negative additive effect (-0.52) confirming that the allele driving lower galling indices originates from the resistant parent, Punjab Jhaar Karela-1. Consequently, *qRKN_pau6.1* represents the major-effect QTL associated with the recessive resistance phenotype.

Conversely, the remaining four minor QTLs (*qRKN_pau3.1*, *qRKN_pau6.2*, *qRKN_pau9.1*, and *qRKN_pau11.1*) may function as genetic modifiers or minor-effect loci. Environmental fluctuations in field screenings likely shift the baseline expression of nematode disease stress. This explains why *qRKN_pau6.2*, *qRKN_pau3.1*, and *qRKN_pau9.1* were exclusively captured in field trials. These modifiers likely modulate the expressivity of the major QTL, regulating downstream physical barriers, chemical signaling, or root system architecture alterations to alter overall galling intensity.

All discovered QTLs displayed negative additive effects, confirming that the resistance alleles originated from the wild parent, Punjab Jhaar Karela-1. Further, the localized genomic polymorphisms flanking the major *qRKN_pau6.1* locus was converted into functional Kompetitive Allele-Specific PCR (KASP) assays. Out of eleven high-efficiency designs, five markers exhibited clear parental polymorphism. Linkage mapping with the markers BGST_19012724 (4.23 cM) and BGST_29234979 (19.05 cM) verified their close proximity to the major resistance locus. The KASP marker BGST_19012724 offer a reliable marker, for screening large germplasm collections, enabling breeders to precisely track and introgress wild-derived *M. balsamina* nematode resistance into commercial elite lines of bitter gourd. Although BGST_29234979 showed linkage with the resistance QTL, its relatively large genetic distance (19.05 cM) may permit considerable recombination between the marker and the causal locus, potentially leading to incorrect selection during MAS. Therefore, this marker should be considered a linked marker rather than a diagnostic marker, and further validation and identification of more closely linked markers are required to improve the accuracy and reliability of MAS.

To further strengthen the biological interpretation of *qRKN_pau6.1*, we annotated the genes present within the KASP-validated region surrounding the marker *BGST_19012724*. Several defense-associated genes were identified, including *Moc06g40420* (MYB), *Moc06g40350* (peroxidase), *Moc06g39650* (bHLH), *Moc06g40190* (receptor-like kinase), and ERF- and protein kinase-related genes, suggesting potential involvement in transcriptional regulation, ROS metabolism, defense signaling and secondary metabolism during *M. incognita* infection (Domínguez-Figueroa et al., 2024). Notably, *Moc06g40420*, encoding a MYB transcription factor, is particularly relevant because tomato *SlMYB108* has recently been demonstrated to contribute to RKN resistance and regulate phenol, flavonoid and lignin accumulation (Luo et al., 2025). The presence of the peroxidase gene *Moc06g40350* further supports a possible role of ROS-mediated defense and lignification. Collectively, these annotations provide additional biological support for *qRKN_pau6.1* and identify putative candidate genes, although functional validation is required to establish their direct contribution to resistance.

Marker validation was performed within the same F_2:3_ population used for QTL identification, rather than in an independent validation population. Therefore, the observed marker-phenotype associations should be considered preliminary, and further validation using independent segregating populations and diverse genetic backgrounds will be needed to confirm the robustness and practical utility of these markers for MAS. Collectively, this high-density linkage map provides a robust foundation for marker-assisted selection (MAS) and the targeted introgression of nematode resistance into elite bitter gourd cultivars.

## 5 Conclusion

This study represents the first successful identification and mapping of root-knot nematode (RKN) resistance loci in bitter gourd, achieving a genetic introgression from *Momordica balsamina* into cultivated *M. charantia*. By using a genotyping-by-sequencing (GBS) approach, a robust linkage map was constructed, and five distinct QTLs governing RKN resistance were identified with one major and four minor effect loci. Furthermore, the conversion of these SNPs into functional KASP markers provides a high-throughput, non-destructive alternative to traditional, seedling-damaging phenotyping. By enabling effective screening at the early nursery stage, these markers establish a valuable asset for marker-assisted selection (MAS) and the accelerated breeding of climate-resilient, RKN-resistant bitter gourd cultivars.

## Data Availability

The raw GBS data supporting this study have been submitted into the Indian Biological Data Centre (IBDC) under the accession number PRJEB110870.
