# Supplementary material for: Identification of Meloidogyne incognita resistance QTLs using high density GBS linkage map in bittergourd
Source: Front Plant Sci. 2026 Sep 23;17:1920925. doi: 10.3389/fpls.2026.1920925 (PMC13642707; doi:10.3389/fpls.2026.1920925)
Supplement: Supplementary file 1 [file Table1.docx]

**Supplementary Table 1. Physical (bp) positions of the reported QTL intervals relative to the reference genome assembly (Urasaki et al., 2017)**

| **Environment** | **QTL name** | **Chromosome** | **QTL position (cM)** | **LOD** | **PVE (%)** | **Left flanking marker** | **Right flanking marker** | **Physical start (bp)** | **Physical end (bp)** | **Physical interval (bp)** | **Physical interval (Mb)** | **CI (cM)** |
| --- | --- | --- | --- | --- | --- | --- | --- | --- | --- | --- | --- | --- |
| Artificial screening, 2024 | *qrkn_pau_6.1* | 6 | 2095.7 | 5.1 | 10.11 | rs.chr6.31007121 | rs.chr6.31856674 | 31,007,121 | 31,856,674 | 849,554 | 0.850 | 17.8 |
| Artificial screening, 2024 | *qrkn_pau_11.1* | 11 | 15.01 | 3.8 | 7.53 | rs.chr11.473048 | rs.chr11.1210310 | 473,048 | 1,210,310 | 737,263 | 0.737 | 14.0 |
| Field screening, 2024 | *qrkn_pau_3.1* | 3 | 483.11 | 4.5 | 9.09 | rs.chr3.5765636 | rs.chr3.23279450 | 5,765,636 | 23,279,450 | 17,513,815 | 17.514 | 0.4 |
| Field screening, 2024 | *qrkn_pau_6.2* | 6 | 1172.11 | 3.3 | 6.49 | rs.chr6.1145894 | rs.chr6.6760768 | 1,145,894 | 6,760,768 | 5,614,875 | 5.615 | 3.3 |
| Field screening, 2024 | *qrkn_pau_9.1* | 9 | 294.21 | 4.6 | 9.16 | rs.chr9.26322477 | rs.chr9.26336372 | 26,322,477 | 26,336,372 | 13,896 | 0.014 | 4.6 |

**Supplementary Table 2: List of KASP primers designed from putative candidate region associated with *qRKN_pau6.1* QTL region for root knot nematode resistance**

| **S. No.** | **Primer ID** | **SNP Position** | **Primer type** | **Primer Sequence** | **Allele** |
| --- | --- | --- | --- | --- | --- |
| **1.** | **BGST_26595558** | 26595558 | Allele Specific (HEX) | GAAAGAACTCACCCTTGC | C |
|  |  |  | Allele Specific (FAM) | GAAAGAACTCACCCTTGT | T |
|  |  |  | Common primer | GCACAGATCATTATAGCTAGG |  |
| **2.** | **BGST_29234979** | 29234979 | Allele Specific (HEX) | TTTAGCACGACCAGCCAC | C |
|  |  |  | Allele Specific (FAM) | TTTAGCACGACCAGCCAT | T |
|  |  |  | Common primer | AAACCGAAACAATGGGACCT |  |
| **3.** | **BGST_31254815** | 31254815 | Allele Specific (HEX) | AGGTAGTGAGTTGAGAAGAAGA | A |
|  |  |  | Allele Specific (FAM) | AGGTAGTGAGTTGAGAAGAAGT | T |
|  |  |  | Common primer | AGGCATATGAGAAGAAAACC |  |
| **4.** | **BGST_27733738** | 27733738 | Allele Specific (HEX) | ACCAGTAGAAGCTTCAGTGTCA | A |
|  |  |  | Allele Specific (FAM) | ACCAGTAGAAGCTTCAGTGTCG | G |
|  |  |  | Common primer | CTGCTTGTCTCTCCCCACT |  |
| **5.** | **BGST_19012724** | 19012724 | Allele Specific (HEX) | GACCTAGCAATATTCTTCGCAA | A |
|  |  |  | Allele Specific (FAM) | GACCTAGCAATATTCTTCGCAG | G |
|  |  |  | Common primer | GTGGACATTTTGCTGGAC |  |
| **6.** | **BGST_31856694** | 31856694 | Allele Specific (HEX) | CAGAAGCAAACAGCAGAAAAAT | T |
|  |  |  | Allele Specific (FAM) | CAGAAGCAAACAGCAGAAAAAG | G |
|  |  |  | Common primer | CAGCAAACAAACGGAGGG |  |
| **7.** | **BGST_20835096** | 20835096 | Allele Specific (HEX) | AAATTTGAGAGAGTGAAGAC | C |
|  |  |  | Allele Specific (FAM) | AAATTTGAGAGAGTGAAGAA | A |
|  |  |  | Common primer | CTTTGGCTCTGAGAAATC |  |
| **8.** | **BGST_22998840** | 22998840 | Allele Specific (HEX) | GCGATTCCACATTATTTAT | T |
|  |  |  | Allele Specific (FAM) | GCGATTCCACATTATTTAG | G |
|  |  |  | Common primer | TCCTGCAAAACACAATCA |  |
| **9.** | **BGST_30088637** | 30088637 | Allele Specific (HEX) | AAAAGAAGCTCCGTGTCT | T |
|  |  |  | Allele Specific (FAM) | AAAAGAAGCTCCGTGTCC | C |
|  |  |  | Common primer | CACTATCTGCGAAGTCTTTT |  |
| **10.** | **BGST_30495211** | 30495211 | Allele Specific (HEX) | GAAAAAATTTAGGCAACACCA | A |
|  |  |  | Allele Specific (FAM) | GAAAAAATTTAGGCAACACCG | G |
|  |  |  | Common primer | CTTTAACCCAGCACATTCCA |  |
| **11.** | **BGST_31011078** | 31011078 | Allele Specific (HEX) | GTTCCAGAGAATCACACC | C |
|  |  |  | Allele Specific (FAM) | GTTCCAGAGAATCACACA | A |
|  |  |  | Common primer | GTGCCCAAGTAGAGGAAG |  |
| **12.** | **BGST_31760159** | 31760159 | Allele Specific (HEX) | AAGCCCATGTTCTACCAT | T |
|  |  |  | Allele Specific (FAM) | AAGCCCATGTTCTACCAC | C |
|  |  |  | Common primer | GAAAGTCCTATCTTCAACCT |  |
| **13.** | **BGST_20102950** | 20102950 | Allele Specific (HEX) | ACTTGACATGGTTCCTTTCT | T |
|  |  |  | Allele Specific (FAM) | ACTTGACATGGTTCCTTTCC | C |
|  |  |  | Common primer | CATGATTAGGGGACCACA |  |
| **14.** | **BGST_21682187** | 21682187 | Allele Specific (HEX) | GGAAGGATGAAAATGAAGA | A |
|  |  |  | Allele Specific (FAM) | GGAAGGATGAAAATGAAGG | G |
|  |  |  | Common primer | GCATCATTTCCATTTTCTTC |  |
| **15.** | **BGST_21665024** | 21665024 | Allele Specific (HEX) | CAATATTGGGTTTAAGGGCATC | C |
|  |  |  | Allele Specific (FAM) | CAATATTGGGTTTAAGGGCATT | T |
|  |  |  | Common primer | CAGCGGTCACATCCTTGAG |  |
| **16.** | **BGST_31011078** | 31011078 | Allele Specific (HEX) | ACGTTCCAGAGAATCACACC | C |
|  |  |  | Allele Specific (FAM) | ACGTTCCAGAGAATCACACA | A |
|  |  |  | Common primer | GTGCCCAAGTAGAGGAAG |  |

**Supplementary Table 3: ANNOVA summary for the markers BGST_19012724 and BGST_29234979**

| **Marker** | **N** | **No. of genotype classes** | **df between** | **df within** | **F** | **P_value** | **Eta squared** |
| --- | --- | --- | --- | --- | --- | --- | --- |
| BGST_19012724 | 166 | 2 | 1 | 164 | 127.4927 | 3.08E-22 | 0.437379 |
| BGST_29234979 | 138 | 2 | 1 | 136 | 0.613471 | 0.434846 | 0.004491 |

**Supplementary Table 4. Putative defense-related candidate genes identified within the *qRKN_pau6.1* QTL region**

| Gene ID | Putative expressed protein | Putative defense-related function |
| --- | --- | --- |
| Moc06g40190 | MDIS1-interacting receptor-like kinase 2-like | Putative pathogen-recognition receptor; may activate immune signalling and downstream defense responses. |
| Moc06g39650 | bHLH126-like transcription factor | Transcriptional regulation of jasmonate-responsive and phenylpropanoid pathways; potentially promotes flavonoid and lignin-associated defense. |
| Moc06g40420 | MYB domain-containing protein-like | Putative regulator of phenylpropanoid, flavonoid and lignin biosynthesis, pathways associated with chemical and structural defense. |
| Moc06g40350 | Peroxidase | Catalyzes ROS-related reactions and lignin cross-linking, contributing to cell-wall strengthening and pathogen defense. |
| Moc06g40100 | Serine/threonine-protein kinase | Putative defense-signalling kinase involved in pathogen recognition and phosphorylation-dependent immune signalling. |
| Moc06g40520 | E3 ubiquitin ligase | Regulates protein stability through ubiquitination and may modulate immune signalling and activation of defense responses. |
| Moc06g40450 | AP2/ERF domain-containing transcription factor | Transcriptional regulator associated with stress and pathogen responses, including ethylene-mediated defense signalling. |
| Moc06g40460 | Ethylene-responsive transcription factor ERF105 | May regulate defense-responsive genes and ethylene-associated signaling during pathogen stress. |
| Moc06g40020 | Amine oxidase | Produces hydrogen peroxide during polyamine catabolism, contributing to ROS signalling and defense responses. |
| Moc06g40030 | Amine oxidase | Potential source of H₂O₂ during polyamine catabolism; may contribute to oxidative signaling and pathogen defense. |
| Moc06g39710 | Cytochrome P450 | Potentially involved in biosynthesis or modification of secondary metabolites associated with plant defense. |
| Moc06g40040 | Cytochrome P450 family 706 | Potential role in secondary-metabolite biosynthesis, including compounds involved in chemical defense. |
| Moc06g40050 | Cytochrome P450 family 706 | Potential role in biosynthesis/modification of defense-related secondary metabolites. |
| Moc06g39760 | Phytocyanin domain-containing protein | Potential role in redox homeostasis and lignin-associated processes during defense. |
| Moc06g40440 | Phytocyanin domain-containing protein | Potentially associated with lignin-related processes and ROS/redox balance during defense. |
| Moc06g39800 | 1-Aminocyclopropane-1-carboxylate synthase 1 | Key enzyme in ethylene biosynthesis; ethylene signalling can contribute to plant responses to pathogen stress. |
| Moc06g40510 | Protein kinase domain-containing protein | Putative component of pathogen-induced signal transduction and MAPK-associated defense signalling. |
| Moc06g40620 | Protein kinase domain-containing protein | Putative defense-signalling protein involved in phosphorylation-mediated pathogen response. |
| Moc06g39820 | Zinc finger A20/AN1 domain-containing stress-associated protein | Stress-associated regulatory protein potentially involved in abiotic and biotic stress responses and defense signalling. |
| Moc06g40600 | NOD26-like intrinsic protein | Aquaporin-family protein potentially involved in H₂O₂ transport and redox signalling during stress responses. |
